# Diagnosis and Psychotherapeutic Needs by Early Maladaptive Schemas in Patients With Inflammatory Bowel Disease

**DOI:** 10.3389/fpsyg.2021.807107

**Published:** 2022-02-09

**Authors:** Cornelia Rada, Dan Gheonea, Cristian George Ţieranu, Denisa Elena Popa

**Affiliations:** ^1^Biomedical Department, “Francisc I. Rainer” Anthropology Institute of the Romanian Academy, Bucharest, Romania; ^2^Gastroenterology Department, University of Medicine and Pharmacy of Craiova, Craiova, Romania; ^3^Gastroenterology Department, University of Medicine and Pharmacy “Carol Davila”, Bucharest, Romania; ^4^Gastroenterology Department, “Elias” Emergency University Hospital, Bucharest, Romania

**Keywords:** inflammatory bowel disease, early maladaptive schemas, hiperervigilance, emotional deprivation, depression, anxiety, chronic diseases, inhibitiony

## Abstract

Inflammatory bowel disease (IBD) is chronic and incurable. Imperious diarrhea, rectal bleeding, fatigue, and weight loss, the main manifestations, cause a decrease in the quality of the patient’s personal and professional life. The objectives of this study were to identify a possible relationship between early maladaptive schemas and disease activity status using logistic regression, to identify the prevalence of early maladaptive schemes in patients and to propose a psychotherapeutic intervention plan. The following were found in a sample of 46 patients aged 16–76 years. An increase in the domain overvigilance and inhibition score had a significant effect (Wald = 6.583, *p* = 0.010), with an increase of 1.137 CI95% [1.031, 1.254] of the risk of the disease being diagnosed as active. High and very high scores were observed for the emotional deprivation scheme (nearly three-quarters) and dependence/incompetence, vulnerability to harm and illness and subjugation schemas (over 80%). The results show that the proposed model could predict and reconfirm the diagnosis; patients have specific psychotherapeutic needs. The therapeutic goal would be to offer care, empathy and protection, to strengthen self-confidence, to make patients realize that they have the ability to cope, to provide permission, encourage the patient to experiment, and guide the patient to express their anger healthily. The therapy scheme’s intervention could lead to increased long-term disease management capacity and, consequently, reduce costs directly and indirectly caused by this condition.

## Introduction

Inflammatory bowel disease (IBD) is mainly comprised of Crohn’s disease (CD) and ulcerative colitis (UC). These are chronic, incurable diseases, sometimes only with longer remissions. Imperious diarrhea, rectal bleeding, fatigue, and weight loss, the main manifestations of IBD, cause a decrease in the quality of the patient’s personal and professional life (Saibil, 2011; Nóbrega et al., 2018).

One of the most difficult problems for both the patient and the treatment is fecal urgency, defined as the patient not being able to wait for 15 min or more to go to the bathroom for bowel movements; it is associated with an increase in biomarkers that indicate inflammation. Studies have shown that the proportion of those with a moderate, severe or very severe level of this urgent need for evacuation varies from over 50% to about 83% generating emotional problems for patients as well as a feeling of captivity at home (Dawwas et al., 2021). The large number of stools associated with cramps are symptoms that indicate disease flare-up. Patients with UC may have 2–3 stools during remission periods of the disease, as compared to flare-ups, when they may need to go to the toilet every hour and even more often (Waljee et al., 2009).

The treatment aims to induce and maintain the remission of the disease. Depending on the stage and location of the disease, the treatment consists of oral or/and enemas of 5-aminosalicylic acid, systemic and local corticosteroids, immunomodulators and biological therapy (Lamb et al., 2019).

All have negative side effects, contraindications and the response to treatment is not always as expected, or may be lost with time (Privitera et al., 2021).

The treatment is mainly aimed at reducing inflammation and bowel damage as well as reducing the number of stools and normal consistency. But in Crohn’s disease with fibrostenotic strictures, the bowel diameter on certain portions along the digestive tract becomes narrow, contributing to difficult defecation and increased risk of intestinal obstruction, perforation, abscess and fistula development. Although the use of biological agents has led to better preservation of the mucosa, up to 70% of CD patients require surgery due to fibrostenotic strictures (Yoo et al., 2020).

About 50% of patients develop extraintestinal manifestations the most common being musculoskeletal, ophthalmic, dermatological, and hepatobiliary disorders (Juillerat et al., 2020).

The European Crohn’s and Colitis Organization (ECCO) and the European Society of Gastrointestinal and Abdominal Radiology (ESGAR) illustrate the diagnostic algorithm for IBD based on a combination of clinical, biochemical, stool, endoscopic, and histological investigations.

The unusually strong immunological response to an invading virus or bacteria that targets the digestive tract is thought to play a role in pathogenesis. IBD can be exacerbated by non-causal aggravating factors such as diet and stress (Torres et al., 2020).

Vulnerability to IBD is due to genetic predisposition, gut microbiome, environment and immune response. The condition starts from inflammation of the intestine and can lead to irreversible intestinal fibrosis, steering treatment goals toward maintaining weight and bone health while avoiding hospitalizations and surgery. However, a high proportion of patients reach the need for intestinal resection, with a temporary or permanent stoma, and most of them will develop anastomotic recurrence. After total proctocolectomy and permanent ileostomy, the risk of clinical recurrence is over 25% after 5 years (Torres et al., 2020).

Patients with IBD are also at high risk of developing colorectal cancer. Death from colorectal cancer has decreased, but the risk of colorectal cancer in these patients with IBD has not decreased for over 35 years, despite the introduction of newer therapies with proven efficacy in active disease control (Milanesi et al., 2019). The onset of cancer depends on several factors, such as the extent and duration of the disease and the family history of colorectal cancer. The age at which colorectal cancer is diagnosed in patients with IBD is lower than that for colorectal cancer unrelated to IBD (Annese et al., 2015). Probably this shows that IBD is becoming common in children, adolescents and young people. The diagnosis of IBD is made by various invasive and noninvasive methods, the most important being fecal calprotectin, C-reactive protein, upper and lower gastrointestinal endoscopy, computed tomography, and magnetic resonance, etc. Clinically, several parameters are used for diagnosis, such as the daily number of stools, the urgency of defecation, incontinence, blood in the stool, rectal bleeding, pulse, hemoglobin, temperature, extraintestinal manifestations (joints, eyes, mouth, skin, perianal), etc. These parameters contribute to indices such as the Ulcerative Colitis Colonoscopic Index of Severity, the Crohn’s Disease Digestive Damage Score, Harvey-Bradshaw Index, Crohn’s Disease Activity Index, and the Perianal Disease Activity Index. An indirect indicator of disease activity is health-related quality of life (HRQoL) (Sturm et al., 2019).

Alatab et al. (2020) analyzed data from 195 countries over the period 1990–2017 and found an increase in the prevalence and spending allocated to IBD. During the analyzed period, mortality decreased, as did the average age of diagnosis. At the level of the European population, approximately 0.2% suffer from IBD. For health care, the costs per patient per year reach EUR 3500 for CD and EUR 2000 for UC. In addition, there are indirect costs of approximately EUR 1900 per patient per year due to the loss of labor productivity (Zhao et al., 2021).

Along with medical therapy evolution, changes in the guidelines for diagnosis, treatment, and monitoring have emerged, but a curative treatment for IBD has yet to be found. Psychology plays an important role in efforts to improve living with IBD, but researchers are not so unanimous in their conclusions. Consider the following.

Compared to medical studies, fewer studies have focused on the psychological assessment and psychotherapy of patients with IBD. The pilot randomized controlled trial initiated by Artom et al. (2017) to identify the usefulness of cognitive behavioral therapy (CBT) in managing fatigue in patients with IBD is useful in this regard. One group set out to conduct CBT sessions for 8 weeks by phone or by Skype. The other group provided information to manage fatigue without a therapist. Self-reporting of parameters related to disease activity was performed every 3 months to a year. Another cognitive-behavioral psychotherapy protocol, combined with hypnosis and emotion regulation therapy for patients with BIC, has been proposed by Paulton and Prevost (2020).

Farrell et al. (2016) made specific improvements to the Memorial Symptom Assessment Scale and then used it on a sample of 247 IBD patients in Ireland. The number of symptoms experienced and the perceived burden were higher in patients with active disease. In descending order, the main symptoms identified were lack of energy, bloating diarrhea, and flatulence. From a psychological point of view, the most common symptoms identified were anxiety, difficulty sleeping, embarrassment, and irritation. As a result, in the evaluation of IBD patients, an extension of the number of evaluated symptoms is necessary, as well as increased attention regarding the states of fatigue and various psychological aspects. Perceived functional disability is associated with depressive and anxiety symptoms (Walter et al., 2016). This means that the investigation and treatment of IBD patients must go beyond medical treatment. In the literature, it has been found that group psychotherapeutic intervention with adolescents with chronic conditions such as cancer, diabetes, asthma, and obesity has given encouraging results in managing symptoms and changing behavior (Plante et al., 2001). Grootenhuis et al. (2009) invited 235 adolescents (12–18 years old) diagnosed with IBD from the Netherlands and the United States to participate in an intervention study and managed to receive the participation of twenty-two children. After the intervention was performed in groups, statistically significant positive effects were found on coping, feelings of competence, physical appearance and body image. In an intervention study on 11 adolescents (12–17 years) with IBD and depression, Szigethy et al. (2004) achieved promising results after performing 12 psychotherapy sessions using CBT techniques.

The mechanisms of interaction between the brain and the body are not well known; however, it is understood that chronic physical illnesses negatively impacts mental state, and in turn, psychological problems also affects physical health. Thompson et al. (2011) conducted a review of the literature on children and adolescents with chronic diseases: diabetes, IBD, cancer, and sickle cell disease. The authors found that in patients with IBD, CBT led to psychological well-being and improved quality of life, but further studies are needed to clarify the duration of the results. Most likely, because adolescents have a personality in training, the positive results regarding psychotherapeutic interventions are somehow more evident. The results of studies on adults in psychotherapy with patients diagnosed with IBD are not so clear.

In a review of the literature that analyzed 67 studies to identify the results of complementary therapies in patients with IBD, Duff et al. (2018) found an improvement in the quality of life through a diet rich in fruits, vegetables, and fiber by practicing low- and moderate-intensity exercises. The main mechanism is that of decreasing inflammation. In 29, studies they performed in the analysis of psychotherapy (cognitive behavioral therapy, hypnosis, stress management), the authors found an improvement in quality of life. However, the results on anxiety, depression and disease activity are limited.

CBT relies on a shift of perspective and a rebalance of thoughts, which can help manage stress in general and the specific stress caused by intestinal disorders. However, McCombie et al. (2016) found that positive results were maintained for less than 6 months after the end of the intervention.

In a 2019 literature review Paulides et al. (2021) concluded that psychotherapy can improve the quality of life of IBD patients. They further stated that additional studies are needed to introduce psychological intervention immediately after diagnosis so that the patient learns good constructive coping strategies throughout life.

One hope may come from schema therapy (ST), which was designed primarily to cover what CBT fails to cover in severe personality disorders: borderline and narcissistic. The effectiveness of the scheme therapy is 2–3 years with this severe personality and 1 year for less severe conditions (Beckley, 2010). Bach et al. (2016) found that of the 25 traits (facets) assessed by the Personality Inventory for DSM-5 (PID-5) (American Psychiatric Association, 2013), 15 were associated with schemas and patterns in schema therapy (ST). As a result, the features of the DSM-5 can be considered expressions of patterns and modes that psychotherapists could use in conceptualizing cases and therapy.

Developed in childhood, adolescence, and throughout life, their role was to ensure survival in the face of various life situations. Due to the need for cognitive consistency, people continue to use the same thought patterns in interpreting life situations, although these patterns no longer fit. They sabotage themselves without realizing that the schemes that acted as coping mechanisms in a certain phase of life may no longer help them. Young et al. (2015) state that harmful experiences with parents or significant caregivers repeated in childhood and adolescence in combination with temperamental factors lead to the formation of pathological thought patterns and dysfunctional behaviors. They are triggered when a current situation has similarities with unpleasant situations of the past, with the reaction being dysfunctional, namely, teaching, avoidance or overcompensation. This perpetuates the pattern. Application of The Young Schema Questionnaire—Short form (YSQ-S3) (Young, 2005) validated on the Romanian population (Trip, 2006) and Schema Mode Inventory (SMI) (Young et al., 2008) is instead used for obtaining discussion material. The depth assessment is performed by working with images and can last for 2–3 months.

The largest basis for evidence of the effectiveness of ST mainly comes from borderline personality disorder patients. However, ST could be useful in other patients in whom the origin of their problems is in childhood or adolescence. Because studies that have focused on psychological evaluation and therapeutic intervention by CBT in patients with BIC have different results, or because the corresponding intervention has had short-term results, this study explores a sample of patients with this condition from the standpoint of early maladaptive schemas.

The objectives of this study are 1. Identification of a possible relationship between early maladaptive schemas and disease activity status by criteria using logistic regression; 2. Identification of the prevalence of early maladaptive schemes in patients with inflammatory bowel disease; 3. Developing a pilot model of psychotherapeutic intervention.

## Materials and Methods

### Participants

Out of all the patients diagnosed with Inflammatory Colon Disease who presented between January 2019 and December 2020 at the gastroenterology departments for evaluation and / or treatment at two hospitals in Craiova and Bucharest, a number of 46 patients were randomly selected. They were asked to participate in the study and after signing the informed consent they were evaluated. 60.9% completed the questionnaires at the hospital guided by a specialist or resident, the remaining 39.1% of the patients completed the questionnaires at home. At the next appointment for the consultation, no later than 1 month the patients brought the questionnaires and the doctor checked the completeness of the answers. The Medical Record containing the most important medical parameters of the patient was completed by the doctor in whose care the patient was. In 10 patients there were inaccuracies, ambiguities in some items from the psychology and omnibus questionnaires that were resolved by telephone by the psychologist.

The national context of evolution of the epidemiological situation determined by the spread of COVID-19 and the massive increase of the number of people infected with the SARS-CoV-2 coronavirus that imposed the declaration on the Romanian territory of the state of emergency and then of the state of alert with its prolongation (Comitetul Naţional pentru Situaţii de Urgenţă [National Committee for Emergency Situations], 2020; Preşedintele României [President of Romania], 2020), determined the team to decide not to continue collecting data in 2021. All measures for quarantine of geographical areas, large number of illnesses, deaths, all restrictions of isolation, social distancing, work at home, difficult entry into hospital for treatment of chronic diseases, etc., world situation at least difficult could to generate a series of emotional problems (Oppenauer et al., 2021) which could have contaminated the answers to the psychological questionnaires.

The basic characteristics of the sample are as follows: with Crohn’s disease (CD), 19 (41.3%), with ulcerative colitis (UC) 27 (58.7%), men 27 (58.7%), women 19 (41.3%) aged between 16 and 76 years (*M* = 41.8; *SD* = 14.95), and the majority (87.0%) were from urban areas. At the endoscopic examinations, 71.7% had some lesions. At the time of research, 60.9% of the subjects were in a state of biological remission and 23.9% underwent ileostoma or colostoma.

### Statistical Analysis

The evaluation of early maladaptive schemas was performed with the Questionnaire of Young Cognitive Schemes, short form 3 (YSQ-S3), validated in Romania in 2006. The structure of the analyzes and discussions in this study is based on the concept of the construction of this questionnaire and the therapy scheme created by the authors which states the existence of 18 schemes which they divided into 5 domains (Young, 2005; Young et al., 2015). For the study’s first objective, the scores were calculated on schemes according to the indications found in the literature (Trip, 2006).

For the second objective of the study, the scores obtained were then used to calculate the following scores (as the sum of the scores of the schemes) for the 5 domains: separation and rejection, autonomy and performance, lack of limits, dependence on others, and hypervigilance and inhibition (Table 1).

**TABLE 1:** Distribution of scores by domain according to disease status.

Domains of early maladaptive schemas	Disease in biological remission		Biologically active disease	
	Average	Standard deviation	Average	Standard deviation
Disconnection and rejection	52.14	17.59	52.06	23.85
Impaired autonomy and performance	42.00	15.75	39.94	15.35
Impaired limits	27.89	9.66	24.94	8.75
Other directedness	66.93	17.81	58.22	15.29
Overvigilance and inhibition	90.64	27.87	91.50	29.49

Given the sample size of only 46 subjects, domain scores were used in regression as independent variables. The logistic model (or logit model) was used to model the probability of the existence of a certain class or event. As a binary model of logistic regression, the dependent variable has two levels (categories). A linear binary logistic regression model was used to identify the relationship between the scores, observed on the 5 domains formed by the 18 dysfunctional cognitive schemes, proposed by Young and the subjects’ health condition (1 = active disease, 0 = remission disease).

The research hypothesis consisted of the assumption that the scores observed on the 18 schemes could give a reasonable indication of the medical condition in the case of people diagnosed with IBD. Following the preliminary analysis of the data to identify the presence of outliers and collinearity problems, the sample was reduced to *N* = 45 subjects after eliminating the cases with standard residues higher than 2. Additionally, out of the five independent variables calculated for the domains, the independent variable representing the separation and rejection domain was removed from the model because the corresponding “B” coefficient in the regression equation was zero.

The final model analyzed was formalized in the binary regression equation:

Logit (p) = B0 + B1 * Impaired Autonomy and Performance + B2 * Impaired Limits + B3 * Other Directedness + B4 * Overvigilance and Inhibition, where p is the probability that the subject will be identified as having active disease.

## Results

### Identification of a Possible Relationship Between the Schemes and the Activity Status of the Disease on Biological Criteria Using Logistic Regression

The regression model analyzed was statistically significant χ^2^(4) = 15.247, *p* = 0.004.

In this study the model value of the Nagelkerke pseudo *R*^2^ indicator for the magnitude of the effect on the variability of the health condition was moderate at 0.391. Nagelkerke’s R-square categories are weak (under 0.3), moderate (0.3–0.6) and strong (value higher than 6) (Garson, 2016).

The model correctly classified 75.6% of cases. The linearity hypothesis between the 4 continuous scores and log odds for the binary response was satisfactorily verified by a Box-Tidwell test.

The Hosmer–Lemeshow test also indicated that the proposed regressive model was well adjusted: χ^2^(7) = 5.688, *p* = 0.577 > 0.05. The test is considered more robust than the traditional chi-square test, especially if the independent variables are continuous or the test involves a small sample size. The presence of extreme values (outliers) and collinearity problems in the model data was examined in SPSS satisfactorily.

For the other directedness domain (which generally indicates dependence on others) and the overvigilance and inhibition domain, the four variable domains represent the scores observed on the dysfunctional cognitive schema domains, the regression model coefficients were significantly different from zero according to Wald tests.

The increase in other directedness scores was associated with a decrease (Wald = 7.404, *p* = 0.007) by a factor of 0.836 CI95% [0.735, 0.951] of the odds that colonic disease would be actively diagnosed. The domain includes the Young schemes: subjugation (SB), self-sacrifice (SS) and approval Seeking/Recognition Seeking (AS).

In contrast, the model indicated that an increase in the overvigilance and inhibition score had a significant effect (Wald = 6.583, *p* = 0.010), with an increase of 1.137 CI95% [1.031, 1.254] of the risk of the disease being diagnosed as active. The domain includes the Young schemes: negativity/pessimism (NP), emotional inhibition (EI), unrealistic standards, i.e., hypercriticalness (US) and punitiveness (PU).

The Table 2 contains the logistic regression coefficients corresponding to the independent variables included in the model.

**TABLE 2:** Logistic regression—variables in the equation and coefficients.

Variables	B	S.E.	Wald	df	Sig.		95% C.I.for EXP(B)	
						Exp(B)	Lower	Upper
Impaired autonomy and performance	–0.043	0.039	1.226	1	0.268	0.958	0.888	1.034
Impaired limits	–0.083	0.079	1.126	1	0.289	0.920	0.789	1.073
Other directedness	–0.179	0.066	7.404	1	0.007	0.836	0.735	0.951
Over-vigilance and inhibition	0.128	0.050	6.583	1	0.010	1.137	1.031	1.254
Constant	2.912	1.704	2.919	1	0.088	18.396	0.888	
								

The model was also verified with Cytel LogXact version 12 (Statistical software Cytel Inc., Cambridge, MA). For the exact methods. Both the exact standard method and the Monte Carlo method indicated that the variables other directedness and overvigilance and inhibition have significant effects in diagnosing the disease as active or, conversely, in remission. The regression model was also validated by the statistics from Cytel LogXact, deviance (44.42, df = 40, *p* = 0.29 > 0.05) and likelihood ratio (17.96, df = 5, *p* = 0.003 < 0.05).

The diagnostic performance of the proposed logistic model or its accuracy of discriminating the cases in which the colonic disease was active compared to the cases in which the disease was in remission (STATUS variable) was evaluated using the analysis in SPSS of the ROC curve (Receiver Operating Characteristic). The performance of the model was indicated by the area under the curve (AUC), whose value was quite close to 1.0 (AUC = 0.805, 95% CI [0.677, 0.932]) and statistically significant (*p* = 0.001).

### Early Maladaptive Schemes in Patients With Inflammatory Bowel Disease

As seen in Table 3, high and very high scores in a proportion of over 60% were recorded in the schemes emotional deprivation, abandonment/instability, mistrust/abuse, social isolation/estrangement (domain disconnection and rejection), dependence/incompetence, vulnerability to harm and illness, enmeshment/underdeveloped self (domain impaired autonomy and performance) subjugation (domain other directedness), negativity/pessimism, and emotional inhibition (domain overvigilance and inhibition).

**TABLE 3:** Distribution of subjects by domains and maladaptive schemes.

Schemes/score level	Low		Medium		Low medium	High		Very high		High very high
	*n*	%	*n*	%	%	*n*	%	*n*	%	%
Emotional deprivation	–	–	12	26.1	26.1	16	34.8	18	39.1	73.9
Abandonment/instability	9	19.6	7	15.2	34.8	14	30.4	16	34.8	65.2
Mistrust/abuse	6	13.0	10	21.7	34.7	13	28.3	17	37.0	65.3
Social isolation/estrangement	10	21.7	7	15.2	36.9	15	32.6	14	30.5	63.1
Defectiveness/shame	–	–	24	52.2	52.2	22	47.8	–	–	47.8
Failure	–	–	20	43.5	43.5	7	15.2	19	41.3	56.5
Dependence/incompetence	3	6.5	14	30.4	36.9	14	30.4	23	50.0	80.4
Vulnerability to harm and illness	–	–	7	15.2	15.2	21	45.7	18	39.1	84.8
Enmeshment/underdeveloped self)	8	17.4	8	17.4	34.8	12	26.1	18	39.1	65.2
(Entitlement/grandiosity	11	23.9	10	21.7	45.6	10	21.7	15	32.7	54.4
Insufficient self-control/self-discipline	13	28.3	11	23.9	52.2	13	28.3	9	19.6	47.9
Subjugation	5	10.9	4	8.7	19.6	17	37.0	20	43.5	80.5
Self-sacrifice	11	23.9	18	39.1	63	10	21.7	7	15.2	36.9
Approval seeking/recognition seeking	8	17.4	15	32.6	50	12	26.1	11	23.9	50
Negativity/pessimism	7	15.2	10	21.8	37	14	30.4	15	32.6	63
Emotional inhibition	6	13.0	8	17.4	30.4	16	34.8	16	34.8	69.6
Unrealistic Standards/hypercriticalness	11	23.9	13	28.3	52.2	10	21.7	12	26.1	47.8
Punitiveness	16	34.8	6	13.0	47.8	12	26.1	12	26.1	52.2

## Discussion

### Regression Model of Early Maladaptive Schemas and Disease Status

The first objective of the study, started from the hypothesis that the scores observed on the 18 schemes could provide a reasonable indication of the medical condition. The hypothesis was confirmed for 8 schemes. Statistically significant results show that the proposed binary logistic regression model predicts with acceptable practical accuracy (75.6%) the evolution status of colonic disease, independent of diagnosis by laboratory analysis. The domains of other directedness, overvigilance and inhibition can be statistically significant predictors of disease status, whether active or in remission. In other words, high scores on the subjugation (SB), self-sacrifice (SS), approval seeking/recognition seeking (AS) schemes may be an indicator of disease remission. High scores on the negativity/pessimism (NP), emotional inhibition (EI), unrealistic standards, hypercriticalness (US), and punitiveness (PU) schemes may be an indicator of active disease.

The fact that these areas and the schemes they cover can provide information about the status of the disease is important because the application of YSQ can be an alternative to evaluation if the patient cannot reach the doctor or cannot do tests. It can be a way to predict and reconfirm the status of the disease but also a means to possibly reduce the costs associated with analyses.

However, the small sample size provided only approximately 10 cases for each of the four independent variables, despite the “rule of 10” (minimum of 10 cases per independent variable) mentioned by Hosmer et al. (2013).

In patients in this study with IBD, the biological activity status of the disease was performed according to the standards, taking into account C Reactive Protein, Erythrocytes Sedimentation Rate and Fecal Calprotectin analyzes, which had high values and indicated inflammation. The fact that the Regression Model indicated that high scores on negativity/pessimism may be an indicator of active disease appears to be valid considering that also Roy et al., 2010) in their study on 6,814 persons aged 45–84 years with no history of clinical cardiovascular disease found that high scores of pessimism were associated with tests indicating high levels of inflammation.

The response to stressed events may be emotional inhibition materialized by shyness, social avoidance, decreased expression of emotions aspects that are explained as a result of arousal, hyperactivity of the limbic-hypothalamic system (Traue et al., 2016). Therefore, the high score on emotional inhibition as an indicator of active IBD identified in this sample can be interpreted as a response to the stress that the disease creates in general and especially when it is active, manifest (uncontrollable, diarrhea, fatigue, fear etc.). At the same time, it can be a coping mechanism through which the patient withdraws from the communication transactions from interactions that he considers demanding, consuming energy.

It is necessary for the patient to be informed that long-term emotional inhibition has negative effects on health and to be encouraged to get out of this unprofitable circle: illness—exaggerated withdrawal—illness. Becker et al. (2015) in a study of 281 IBD patients and 32 family members over 60% reported impairment of agreement activities and over 50% reported impairment of interpersonal relationships. So it is about a social avoidance that can be interpreted as both the cause and the effect of the disease activity.

Unrealistic standards, hypercriticalness in relation to oneself and others, denote according to Young et al. (2015) a deficit regarding the feeling of pleasure, relaxation, health, self-esteem, relationships, etc. The fact that high scores on this scheme may be an indicator of active disease is related to high scores on the previous two schemes (Negativity/pessimism and emotional inhibition). The question is how could someone pessimistic, emotionally inhibited to relax especially, if it is mostly driven by perfectionism, rigid rules, the idea of performance and efficiency? It would probably be very difficult. It is possible that the patient in remission to increase these characteristics of the scheme in order to recover what he fails to manage when it is in the stage of active disease, but this mode leads him to relapse by overload. The patient must be taught to adjust his expectations regarding work, friends, marriage and helped to accept that this condition involves only a loss to which it is useful for health to adapt otherwise it creates a psychological distress. Like Sirois et al. (2021), who found associations between perfectionism and health problems in people with chronic fatigue syndrome, the present study on the IBD sample indicates the need to understand the health risks of unrealistic standards in the context of chronic diseases.

Several studies are needed to see why high scores on the punitiveness scheme are an indicator of active disease as well as to identify how it manifests itself: by capitulation, avoidance or overcompensation. For what reasons is this scheme activated, which implies the difficulty in forgiving the mistakes of oneself and others, the failure to take into account the attenuated circumstances and a low manifestation of empathy at the emotional level? (Farrell et al., 2014). Being from the cluster of the hypervigilance domain is related to the other three schemes mentioned above. It needs to be clarified whether it is a punishment because they feel deficient or a kind of internalization of the punitive parent from their childhood. Whatever the causes and manifestations of this scheme, the patient must learn healthy indulgence and get rid of that critical, harsh parental voice from which he is in danger of entering the active phase of the disease.

### Early Maladaptive Schemas

Regarding the second objective of the study, identification of the prevalence of early maladaptive schemes in patients with IBD, as a result of the small sample size, discussions will focus on those schemes with high and very high-level scores, namely, an emotional deprivation score of almost 80% and dependence/incompetence, vulnerability to harm and illness schemes and subjugation scores in proportions of over 80%.

Almost three-quarters of the sample had high and very high scores on the emotional deprivation scheme. Patients with BIC in this sample tend to believe that the need for an emotional connection is not being met or will not be provided.

Nearly three-quarters of the sample had high and very high scores on the emotional deprivation scheme. Patients with BIC in this sample tend to believe that their need for emotional connection will not be met. Here, the specialist can intervene by offering (1) care, (2) empathy, and (3) protection. A scheme belonging to the disconnection and rejection domain separation and respite has an essential role in the therapeutic relationship to provide the patient with affection, warmth and attention. Covering these needs is essential for IBD patients.

In the domain of impaired autonomy and performance, the dependence/incompetence and vulnerability to harm and illness schemes recorded high and very high scores over three-quarters of the sample.

Patients with the dependence/incompetence cognitive schema tend to feel that they cannot cope with daily activities without help. Either they are passive or they reach generalized helplessness (Young et al., 2015). The danger of depression may also arise here. Overcompensation can be manifested because they do not ask anyone for help and end up working too hard. Intervention is needed to strengthen self-confidence and to realize the benefit of applying the method of trial and error to the detriment of disregarding one’s person.

Vulnerability to harm and illness is a scheme that can raise major problems in patients with chronic conditions such as IBD. They tend to be anxious that they may suffer an illness, an accident, or that something bad will happen to them (Young, 2008).

In the context of such a condition, which is disabling, it is easy to understand catastrophic thinking about any digestive symptoms, such as bloating and soft stools that are commonplace or circumstantial for others.

Diarrhea, the main obvious manifestation of BIC until the loss of control of the anal sphincter, creates negative emotions that have roots on an unconscious nature as a major loss of an acquisition.

This regards the period when the child was first potty-trained. When the child acquires this habit of controlling the sphincters, which occurs in the interval of 12–24 months, with an approximate average of 18 months (Kiddoo, 2012), all the adults applaud and validate this new behavior. The scheme of vulnerability to harm and illness makes the patient avoid any situation that causes insecurity; in this case, the patient isolates himself in the house as much as possible due to fear of not having access to a toilet. In the long term, they may lose opportunities for fun, relaxation, socialization, and even activities and behaviors that could stimulate the vagus nerve, which has an anti-inflammatory effect (Breit et al., 2018). The therapeutic goal would be to make patients realize that they have the ability to cope. Young et al. (2015) created the concept of the healthy adult mode, which is the healthy, adult part of the self. Therefore, it is necessary to activate this mode to provide support, understanding, and encouragement to the vulnerable child mode in which patients frequently find themselves.

From the other-directedness domain, the subjugation scheme was registered with high and very high scores in a proportion of over 80%. Young et al. (2015) describe patients with this scheme as allowing others to dominate them due to fear of punishment. By subordinating emotions and needs, they often end up accumulating anger that can manifest as uncontrolled outbursts of psychosomatic symptoms. The therapeutic goal would be to provide permission, encourage the patient to experiment, and guide the patient to express their anger healthily.

### Developing a Pilot Model of Psychotherapeutic Intervention

The third objective of the study takes into account the fact that in the absence of sufficient data on the usefulness of therapy focused on early maladaptive schemas, it would be helpful to conduct a pilot study on patients with IBD with the following steps.

1. Recruitment of 30 patients with IBD.
2. Evaluation of patients with an omnibus questionnaire with sociodemographic data, sex life, sleep quality, and physical activity.
3. Collection of patients’ medical data from the records in the hospital where they are in the records.
4. Evaluation of patients through The Young Scheme Questionnaire—Short form (YSQ-S3) and Scheme Mode Inventory (SMI).
5. All patients will benefit from 2 group sessions, one per week, of 50 min, presenting theoretical elements about ST (schemes, modes) and coping mechanisms. Each group will consist of 5 patients.
4. Psychotherapeutic intervention will be performed on half of the patients. They will be divided into groups of 4 according to the identified common coping schemes and mechanisms. One session per week, by 50 min for 6 months (16 sessions per month).

In the evaluation phase, we will work for 2–3 months with images. In the change phase, the emphasis will be on emotions, images, and role-playing games to determine the emotions they feel during therapy. At the same time, CBT techniques and repairers will be used. Young (2008) appreciates that if there is no personality disorder (ex-narcissistic, borderline), then at least 1 year of intervention is needed (2008). Given that it is generally difficult to keep a patient in psychotherapy for so long, and it is only a pilot study, exploring the usefulness of scheme therapy intervention will take only 6 months.

5. After the 6 months of intervention with 10–15 of the patients with whom psychotherapy was done, interviews will be conducted to obtain a clearer perspective of patients on psychotherapeutic intervention, the result obtained and their experience in the therapeutic process. This will make it possible to improve the intervention process.

Evaluation of sleep-related data, self-reported symptoms and specific questionnaires will be performed at the beginning of the study and then at 3 and 6 months.

The application process of the questionnaires will take place online or in the hospitals where it is registered. The therapeutic process will take place on Skype, involving a weekly session of 50 min (2–3 months evaluation, 6 months intervention). If a patient is unable to attend a group psychotherapy session, then the session can be retrieved by telephone. The effectiveness of ST will be evaluated at 3 and 6 months by the patient self-reporting symptoms and the researcher applying tools to assess the change in maladaptive mechanisms of behavioral coping and thinking. At the same time, the medical parameters from the patients’ medical records will be analyzed. This evaluation at 6 months, 3 months after the end of the psychotherapeutic intervention, will allow us to observe the persistence of the positive results over time. Medical parameters and self-report of symptoms will be compared with the group that did not benefit from therapeutic intervention. Regarding the schemes, the aim is for the patients to identify their dysfunctional life patterns, when the maladaptive schemes are triggered and to understand the origin of those schemes in childhood and/or adolescence (Young et al., 2015).

Regarding the modes, the objectives are for the patient to learn ways to overcome their negative patterns of thinking, to identify the modes of child, parent, coping and healthy adult, to work to overcome the maladaptive modes (vulnerable child, angry child, impulsive child, parent demanding on performance, parent inducing guilt parent and punitive parent) and dysfunctional coping modes (capitulation, avoidance, overcompensation) as well as to learn to access the happy child and healthy adult mode (Jacob et al., 2019). By gradually weakening the dysfunctional parts of the personality structure and consolidating the healthy adult part, longer-term changes could occur.

## Conclusion

It cannot be stated with confidence that these identified maladaptive schemes are specific to patients with BIC, making it possible to identify them in other people with chronic diseases such as cardiovascular diseases, diabetes, osteoarticular diseases, neoplasms, etc. However, starting from here, one could identify the specific needs that can serve as a starting point in psychotherapeutic interventions and identify, manage, and support them within a specialized framework. This would represent a change of depth that could influence the way that they think about themselves, others and even about life. Overcoming these schemes could also lead to self-therapy, which could lead to the gradual weakening of the dysfunctional parts of the personality structure.

Exploring scores in the areas of other directedness, overvigilance and inhibition can be a way to validate a diagnosis obtained through medical parameters, but it could especially serve as an inexpensive way to identify whether the disease is active or in remission.

Higher scores on the negativity/pessimism (NP), emotional inhibition (EI), unrealistic standards/hypocriticalness (US), and punitiveness (PU) schemes could denote active disease status. The main manifestations can be an exaggerated orientation toward negative aspects, avoiding the expression of emotions, and striving to reach high personal standards and rigid rules on efficiency.

Higher scores on subjugation (SB), self-sacrifice (SS), and approval seeking/recognition Seeking (AS) may indicate that the disease is in remission. Manifestations that may denote remission are the orientation of behaviors, emotions, pursuing the needs toward others to the detriment of oneself, emphasizing satisfying the need for approval, recognition and attention from others.

ST focused on overcoming early negative thought patterns could represent hope for deeper and longer-lasting changes in patients with IBD. Patients can learn to recognize their patterns, cure them, and build healthy patterns to better manage their condition, especially in the prevention of relapses.

The fact that ST places much emphasis on emotional techniques and reaches the origins of childhood needs in attachment theory and then reaches CBT techniques such as evidence analysis, providing recommendations and behavioral strategies (Young, 2008) could result not only in a reduction in symptoms but also in a profound change in personality. Intimacy and relationship problems are deep, and as a result would be better suited to ST because, as stated by Young (2008), “When we deal with intimacy issues or relationship problems, they go very deep, and most of us don’t have cognitive awareness of why we are having these issues or how to solve them. This is why CBT doesn’t work very well with characterological issues, in my opinion.”

Without the neutrality of psychoanalysis, with a more flexible structure than CBT, ST can be useful to patients with IBD because it provides warmth and care and allows the therapist to show these feelings to the patient, which leads to a reparative connection.

Of course, the study has some limitations; the main ones are the small sample size, the lack of in-depth anamnesis, such as a life story (personal biography notes), in which to capture the critical moments that led to their formation, and the lack of a control group to make comparisons.

The main limitation of the study is the small sample size. However, it should be noted that the prevalence of IBD is low compared to other chronic diseases. For example in Europe the prevalence per 100,000 persons of CD varies from 1.5 to 213 cases and of UC varies from 2.4 to 294 cases (Burisch et al., 2013) while the prevalence of diabetes in the European Region has an average of 9,200 cases per 100,000 people (International Diabetes Federation, 2021).

The second limitation of the study is that there was no control group. The extension of the state of alert declared in October 2021 in Romania as a result of the pandemic, media presentations of world dramatic situations could not make it possible to set up an authentic control group because a large part of the population could have had some emotional problems (Knolle et al., 2021; Vancea and Apostol, 2021).

The article did not aim to determine the conclusions of the studied subject, namely early maladaptive schemes in patients with IBD, nor was it possible given that this is the first time the subject is approached from this point of view. It tends to be an exploratory study providing a starting point for further investigation. However, some conclusions can be drawn, (1) some of these patients can be helped by psychotherapy (2) knowledge of their own patterns can help patients recognize when the early maladaptive schemes is triggered (3) to do self-therapy focused on covering the needs behind of personal maladaptive schemes. Of course, the predictive value of the realized statistical model is limited due to the small sample. However, this model may indicate how disease recurrences trigger certain maladaptive schemes. At the same time, it offers an extra knowledge regarding the approach of the patient by the doctor and by the psychotherapists from the perspective of the schemes.

The perspective of treating patients with BIC must be that of holistic, multidisciplinary, psychological interventions, either in general or in psychotherapy, and focused on early maladaptive schemas that can lead to significant improvements not only in the short term (during psychotherapy and immediately after) but also in the medium and long term.

Psychological comorbidities constitute a special category that requires review by a gastroenterologist. This could change the course of the disease, increase the quality of life and consequently reduce health care costs.

## Data Availability Statement

The datasets analyzed during the current study are available from the corresponding author on reasonable request.

## Ethics Statement

The study followed the Declaration of Helsinki to grant respect for human rights among all study participants during all phases of the study. Based on the submitted documentation, the Ethics Commission of “Francisc I. Rainer” Anthropology Institute of the Romanian Academy, Bucharest, Romania, issued the approval decision no. 55/23-01-2019. The patients/participants provided their written informed consent to participate in this study.

## Author Contributions

CR performed the statistical analysis. All authors contributed equally to this study regarding data collection, conception, design, material preparation, draft of the manuscript, read, and approved the final manuscript.

## Conflict of Interest

The authors declare that the research was conducted in the absence of any commercial or financial relationships that could be construed as a potential conflict of interest.

## Publisher’s Note

All claims expressed in this article are solely those of the authors and do not necessarily represent those of their affiliated organizations, or those of the publisher, the editors and the reviewers. Any product that may be evaluated in this article, or claim that may be made by its manufacturer, is not guaranteed or endorsed by the publisher.
